# Green Constant-wavelength Concurrent Selective Fluorescence Method for Assay of Ibuprofen and Chlorzoxazone in Presence of Chlorzoxazone Degradation Product

**DOI:** 10.1007/s10895-023-03175-6

**Published:** 2023-02-21

**Authors:** Nora A. Abdallah, Mona E. Fathy, Manar M. Tolba, Amina M. El-Brashy, Fawzia A. Ibrahim

**Affiliations:** Department of Pharmaceutical Analytical Chemistry, Faculty of Pharmacy, Mansoura University, Mansoura, 35516 Egypt

**Keywords:** Spectrofluorimetry, Chlorzoxazone, Ibuprofen, 2-amino 4-chlorophenol, Chlorzoxazone impurity, Pharmaceutical preparations and biological fluids, Greenness assessment tools

## Abstract

Lower back pain is a universal dilemma leaving a negative effect on both health and life quality. It was found that a fixed dose combination of chlorzoxazone and ibuprofen gave a higher efficiency than analgesic alone in treatment of acute lower back pain. Based on the significant benefit of that combination, a green, sensitive, rapid, direct, and cost-effective method is created for concurrent determination of ibuprofen and chlorzoxazone in presence of 2-amino para chlorophenol (a synthetic precursor and potential impurity of chlorzoxazone) adopting the synchronous spectrofluorimetric technique. Synchronous spectrofluorimetric technique is adopted to avoid the highly overlapped native spectra of both drugs. The synchronous spectrofluorometric method was applied at Δλ = 50 nm, ibuprofen was measured at 227 nm while chlorzoxazone was measured at 282 nm with no hindering from one to another. The various experimental variables affecting the performance of the suggested technique were explored and adjusted. The suggested technique showed good linearity from 0.02 to 0.6 and 0.1 to 5.0 µg/mL for ibuprofen and chlorzoxazone, respectively. The produced detection limits were 0.27 × 10^–3^ and 0.03, while the quantitation limits were 0.82 × 10^–3^ and 0.09 µg/mL for ibuprofen and chlorzoxazone, respectively. The suggested approach was successfully applied for the analysis of the studied drugs in the synthetic mixture, different pharmaceutical preparations, and spiked human plasma. The suggested technique was validated with respect to the International Council of Harmonization (ICH) recommendations. The suggested technique was found to be simpler and greener with lower cost compared to the earlier reported methods which required complicated techniques, longer time of analysis, and less safe solvents and reagents. Green profile assessment for the developed method compared with the reported spectrofluorometric method was performed using four assessment tools. These tools confirmed that the recommended technique attained the most possible green parameters, so it could be used as a greener option in routine quality control for analyzing the two drugs in genuine form and pharmaceutical preparations.

## Introduction

Spectrofluorimetry is a very important analytical technique due to its low cost, high selectivity and sensitivity that allow easy quantitative analysis of a lot of pharmaceutical compounds [1–6]. Despite all these great features, it is not useful in the cases of concurrent determination of two drugs that have overlapped emission spectra. To overcome the selectivity problem and maintain the remarkable advantages of spectrofluorimetry, synchronous spectrofluorimetry (SS) was developed by only some careful optimization of the instrumental parameters that allows resolution of that interference of spectra with no pre-separation steps [7]. Reduction of light scattering and simplification of the spectra are the most important feature of SS, that greatly improves the selectivity and sensitivity [8]. It is also characterized by very good selectivity during the analysis of drugs in biological fluids or combined dosage forms due to the high level of tolerance to unrelated substances. Furthermore, the resulted sharp and narrow peaks allows the easy analysis of the drugs [7].

Constant-wavelength synchronous spectrofluorimetry (CWSS) is a category of fluorimetric analysis that depends on scanning the excitation and the emission monochromators concurrently with a constant wavelength difference (Δλ) between them. It is one of the most useful and common types of analysis due to its simplicity, relatively low cost and high sensitivity. The principle, procedure and application of synchronous spectrofluorimetry were reported by Andrade-Eiroa et al. [9].

Low back pain (LBP) is a universal problem that usually appear in the third decade of life. LBP has a very bad effect on general health that affects the life quality and the financial side of the patients, as it limits their ability to work plus their need to spend money on healthcare. LBP is usually linked with muscle cramp, so a fixed dose combination of a muscle relaxant such as chlorzoxazone and a NSAID like ibuprofen was proven to be more effective than ibuprofen monotherapy in treating acute low back pain [10].

**Fig. 1:**
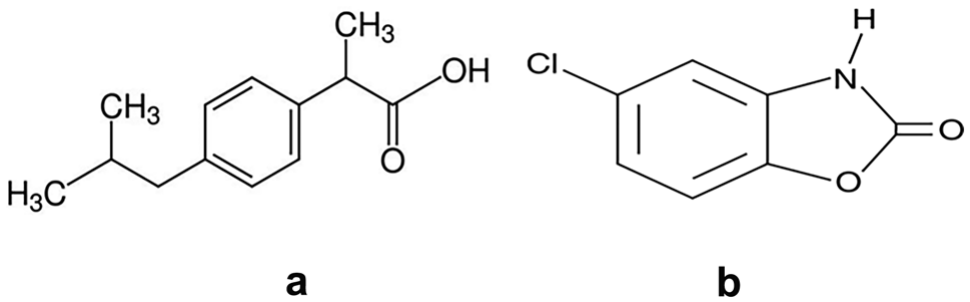
Chemical Structure of **a** IBN **b** CLX

Ibuprofen (IBN in Fig. 1a, is chemically designated as 2-(4-Isobutylphenyl)propionic acid [11]. It is an NSAID that is used in the management of mild to moderate pain and inflammation. It is official in USP [12] which recommend HPLC for its analysis.

Chlorzoxazone (CLX) in Fig. 1b, is acting centrally as skeletal muscle relaxant with some sedative actions. It is claimed to inhibit muscle spasm by applying the primary effect on the level of the spinal cord and subcortical areas of the brain. It is used along with NSAID for symptomatic treatment of painful muscle spasm associated with musculoskeletal conditions. CLX is also co-formulated with different types of analgesics in combined preparations [11]. It is official in USP [12] and it was assayed in it by spectrophotometry in pure form while by HPLC in tablets.

2-amino-4-chlorophenol (ACP) is a synthetic precursor and degradation product of CLX according to USP [7]. It is produced from the alkaline hydrolysis of CLX. It should not be present in marketed formulations above specified limit (< 0.5% as per USP).

From the literature survey, several spectrophotometric methods were published for simultaneous determination of IBN and CLX in tablets or synthetic mixture [13–17]. Three HPLC methods were reported for analysis of that combination in pharmaceutical preparations [18, 19]. An HPLC method for simultaneous determination of IBN, CLX and ACP was reported [20]. Only one spectrofluorimetric method was reported [21] and it depended on second derivative fluorescence spectroscopy.

Our aim was to develop a simpler, more sensitive and greener spectrofluorimetric method for analysis of that important binary combination in both different pharmaceutical preparation and spiked human plasma by taking the advantage of CWSS. The usage of that technique allows direct and concurrent determination of that highly overlapped mixture without any pre- or post- treatment steps. The developed method was also able to detect both drugs in presence of 2-amino para chlorophenol, a degradation product of CLX. The suggested technique was found to be a simple, sensitive and green, which makes it superior to other high-cost/sophisticated techniques.

## Experimental

### Instrumentation

- Agilent G8900A Cary Eclipse Spectrofluorimeter (Agilent, California, USA) was used for the spectrofluorimetric measurements. The synchronous mode was adjusted at Δλ = 50 nm, 20 was adjusted for smoothing the resulting spectra.
- Consort pH-meter was used for pH adjustment.
- The used Vortex mixer was IVM-300P, from Taiwan, while the centrifuge (model 2-16P, SIGMA) was from Germany.

### Materials

- All reagents were of analytical grade and were used with no further purification.
- Authentic samples of IBN and CLX with a purity of 99.66 and 100.81%, respectively were kindly provided by Eva-Pharma Co. Cairo, Egypt.
- Mark-fast^®^ capsules are product of Marcyrl Pharmaceutical Industries, Cairo, Egypt and Myofen^®^ capsules are product of Eva Pharma, Cairo, Egypt. Both dosage forms contain 250 mg CLX and 200 mg IBN/ capsule and were bought from local Egyptian pharmacy.
- Human plasma samples were given by Mansoura University Hospitals (Mansoura, Egypt) and kept frozen at—80 until usage after gentle thawing at room temperature.
- Double distilled water was utilized during the experiments.
- Britton Robinson buffer solutions were used for studying pH effect within range of 2.0–10.0.
- Surfactant solutions with concentration of 1.0% w/v or v/v were prepared in double distilled water then used.
- 2-Amino-4-chlorophenol (ACP): prepared by refluxing 0.25 g of chlorzoxazone bulk powder with 25 ml of 6 M sodium hydroxide for 2 h then cooling and neutralizing using 25 ml 6 M hydrochloric acid. 2-Amino-4-chlorophenol was extracted with diethyl ether and dried under vacuum at 60 °C [22].

### Preparation of Standard Solutions

Stock solutions (200.0 μg/mL) of IBN and CLX were made by dissolving 0.01 gm of each drug in 50.0 mL of ethanol in two separate volumetric flasks [23]. Stock solution (200 μg/mL) of ACP was prepared in ethanol. Working solutions of the two drugs were prepared as appropriate. Both stock and working solution were stored in the refrigerator and were stable for at least one week.

## General Procedures

### Calibration Curve Construction

Different measured volumes of standard solutions of IBN and CLX were transferred into two sets of 10.0 mL volumetric flasks covering a concentration range of 0.02–0.6 and 0.1–5.0 μg/mL for IBN and CLX, respectively. All flasks were completed to the full volume with double distilled water. Subsequently, Synchronous fluorescence spectra were recorded at a steady Δλ of 50 nm. IBN was measured at 227 nm, while CLX was measured at 282 nm. Blank samples were treated at the same manner. Finally, the relative synchronous fluorescence intensity (RSFI) was plotted against the concentration of each drug in μg/mL to obtain the calibration curves and derivatize the corresponding regression equations.

### Synthetic Mixture Analysis

In order to prepare synthetic mixture, different aliquots of working standard solutions of IBN and CLX within the linearity range of each drug and in ratio of their capsules (1:1.25) were transferred into six 10.0 ml volumetric flasks. Double distilled water was used to complete the flasks to the mark. Then, the procedure under "Calibration Curve Construction" was followed.

### Analysis of IBN and CLX in Capsules

Ten Mark fast^®^ or Myofen^®^ capsules were separately evacuated and weighed. An accurate amount equivalent to one capsule content was dissolved in 60 ml of ethanol in two 100.0 ml volumetric flasks. For mixing, sonication was introduced for about ten min, then ethanol was added to full volume followed by filtration. Different volumes of the filtrate within the linear range of each drug were moved into a set of 10 mL volumetric flasks, then the procedure under "Calibration Curve Construction" was followed. From the corresponding regression equations, the nominal contents of the capsules were calculated.

### Analysis of IBN and CLX in Spiked Human Plasma

Using micropipette, one mL of human plasma was transferred into six 15.0 ml centrifugation tubes. Different volumes from IBN and CLX stock solutions within the linearity range were added to the centrifugation tubes to prepare five mixtures with the same capsule ratio (1:1.25). The sixth tube was for the blank determination. All the tubes were subjected to vortex for 3 min then methanol was added as a precipitating agent to make final volume of 5 mLs. Centrifugation was carried out at 4800 rpm for half an hour to complete precipitation. From each tube, the clear supernatant was filtered by syringe filters (0.45 µm) then one mL of the filtrate was withdrawn using micropipette into six 10.0 mL volumetric flasks. Each flask contents were treated as described under "Calibration Curve Construction". The percentage recoveries of each drug and the regression equations were obtained by plotting the fluorescence intensities against the drug concentrations in μg/mL.

### Analysis of IBN and CLX in Presence of 2-amino Para Chlorophenol

Volumes of IBN and CLX, within the linearity range, and volume of ACP were transferred into 10.0 ml volumetric flask to give final concentration of 0.2, 3 and 0.3 μg/mL of IBN, CLX and ACP, respectively. The method was modified using ethanol as diluent instead of water to allow separation of ACP. Then, the procedure was completed as under "Calibration Curve Construction".

## Results and Discussion

### Spectrum Characterization

**Fig. 2:**
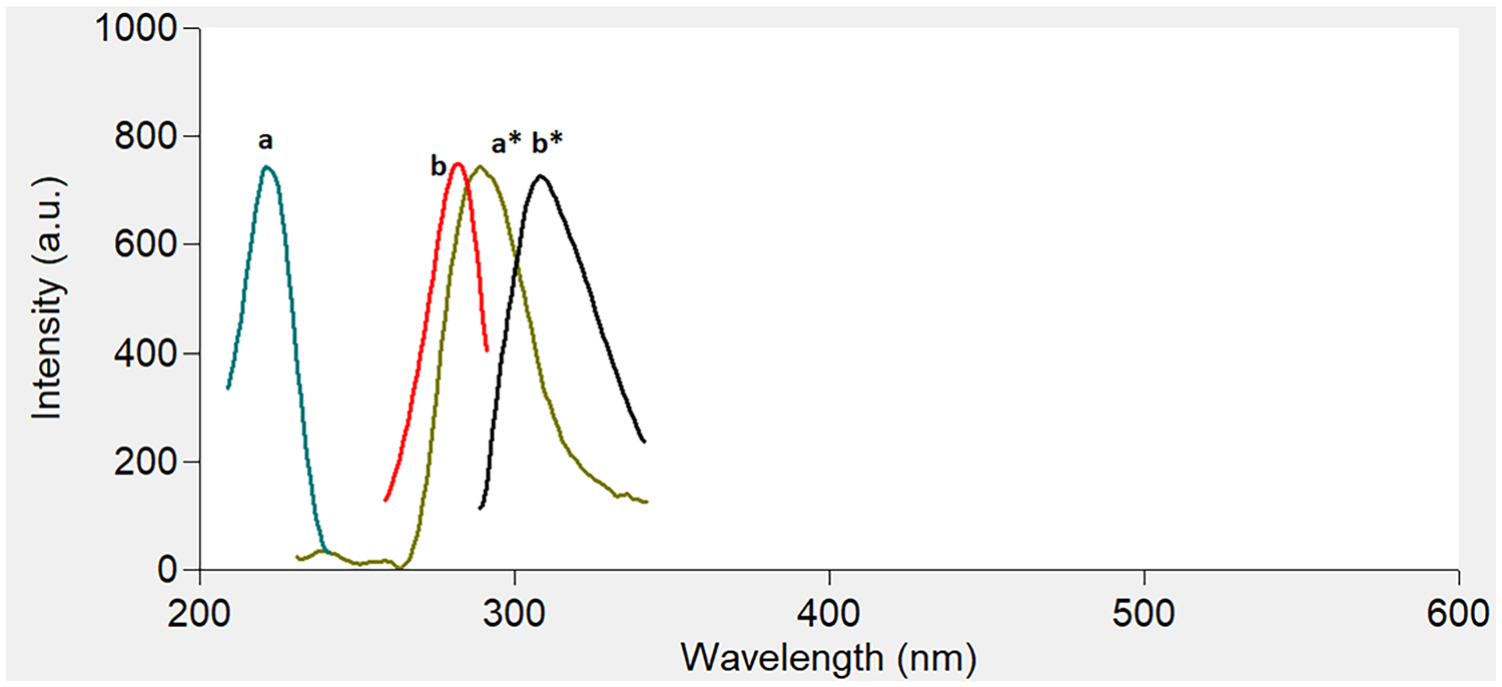
Overlapped excitation and emission spectra of 0.2 μg/ mL of IBN (**a**,a*) and 2.0 μg/ mL CLX (**b**,b*) ethanolic solution

**Fig. 3:**
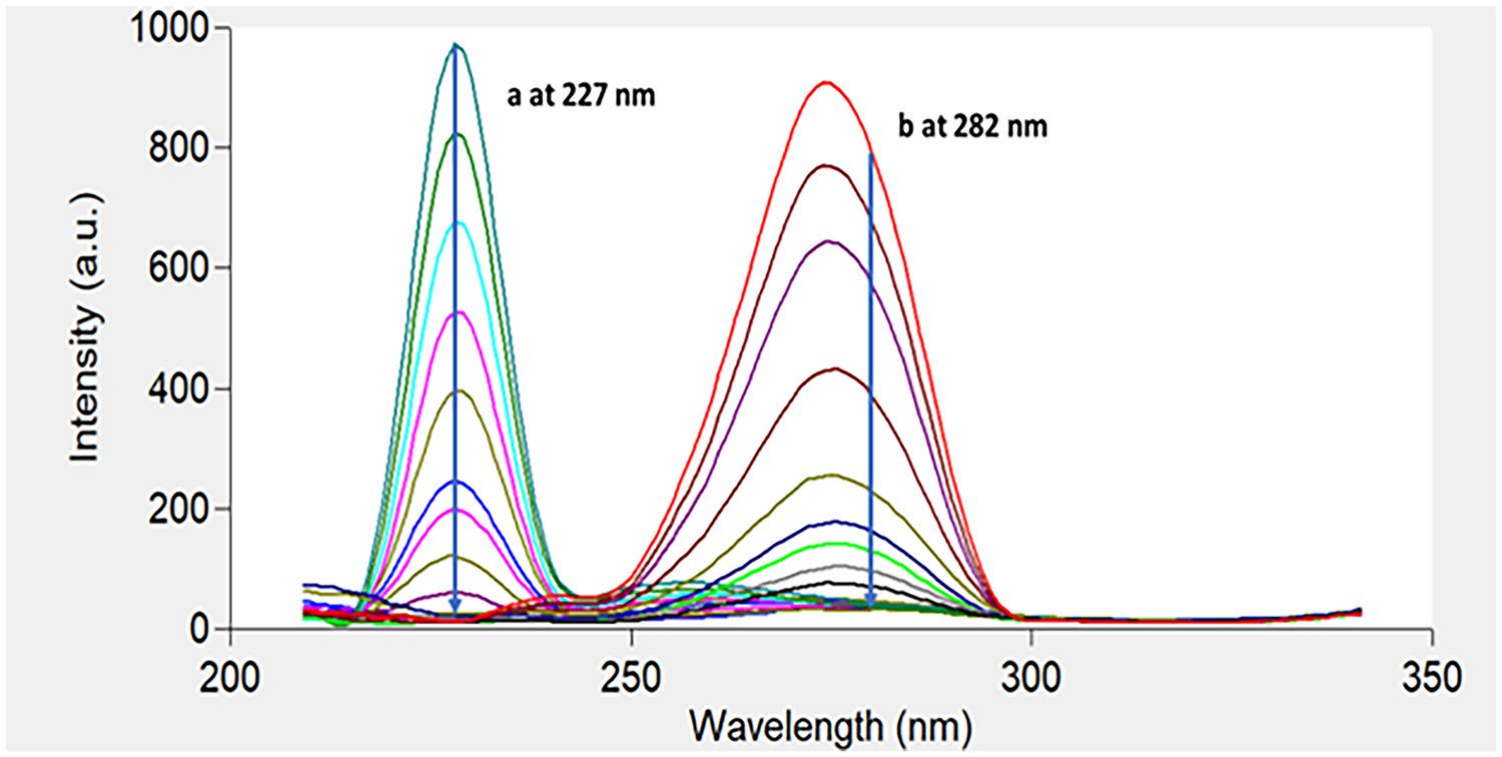
Synchronous Fluorescence spectra of different concentrations of **a** IBN and **b** CLX at Δλ = 50 nm in water

**Fig. 4:**
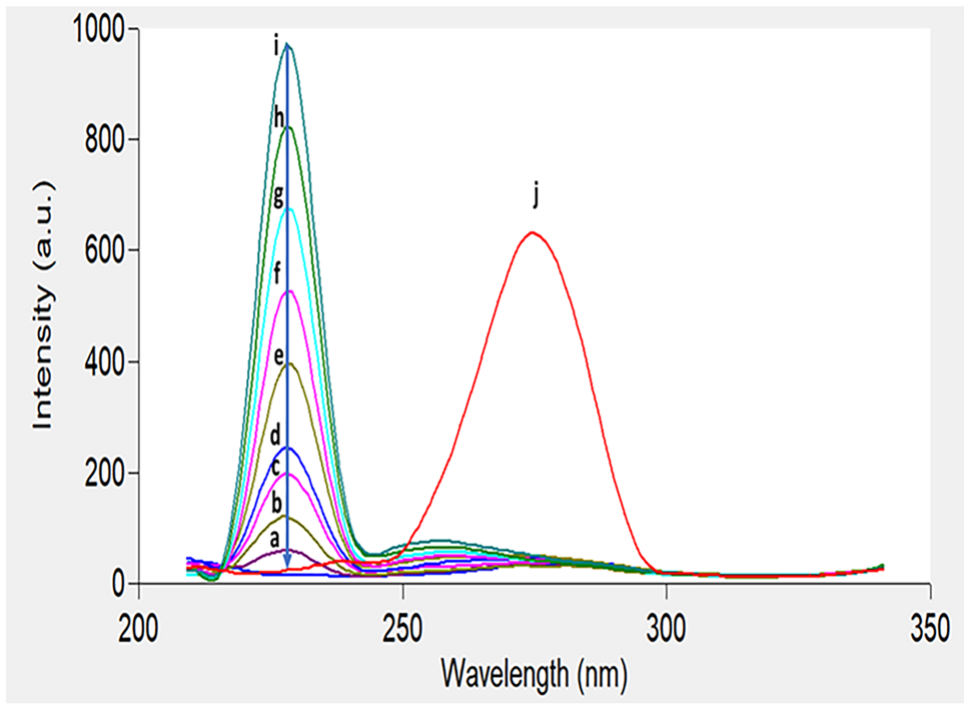
Synchronous fluorescence spectra of (j) CLX (3.0 μg/mL) and (2) IBN (a-i; 0.02, 0.03, 0.05, 0.08, 0.1, 0.2, 0.3, 0.4, 0.5 and 0.6 μg/mL) at 227 nm

**Fig. 5:**
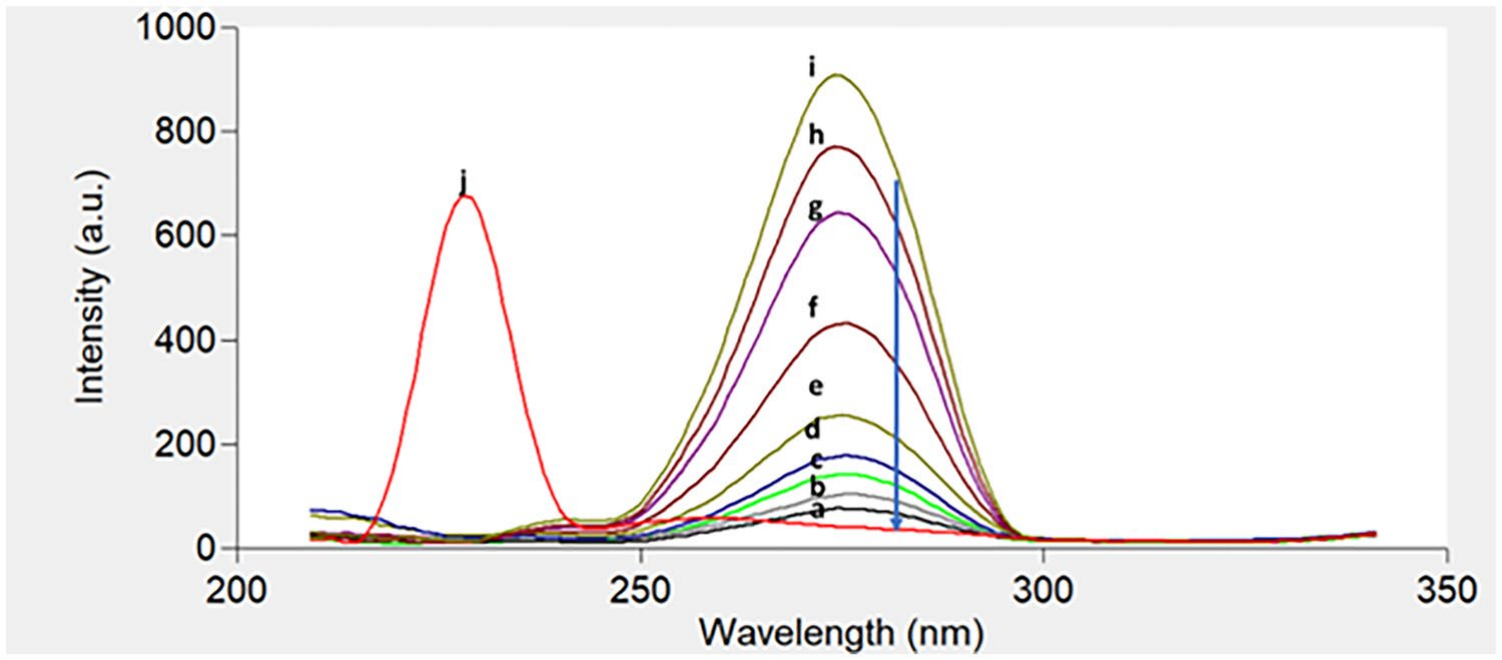
Synchronous fluorescence spectra of (j) IBN (0.4 μg/mL) and (2) CLX (a-i; 0.1, 0.3, 0.5, 0.6, 1.0, 2.0, 3.0, 4.0 and 5.0 μg/mL) at 282 nm

Figure 2 shows an excitation–emission fluorescence spectra of ethanolic solution of IBN and CLX. IBN exhibits strong fluorescence intensity at 289 nm after excitation at 230 nm, while CLX has a fluorescence intensity at 309 nm following excitation at 280 nm. Those spectra were highly overlapped in a behavior that hinders their simultaneous determination especially when it is required to assess the studied drugs in their co-formulated dosage form or in biological matrices. Accordingly, the CWSS technique was the perfect choice for simultaneous determination of that binary mixture in one step. Several Δ λ were tried in range of 10- 140 nm and well resolved peaks were obtained at Δ λ = 50 nm with a zero- crossing point that made easy determination of the two drugs in the presence of each other with no interference. IBN was determined by measuring the intensities of SF spectra at 227 nm, while CLX was determined at 282 nm, as shown in Fig. 3. Figures 4 and 5 showed the SF spectra of various concentrations of either IBN or CLX in the presence of one concentration of the other, upon applying CWSS method using Δ λ = 50 nm.

#### Studying and Optimization of Experimental Factors

**Fig. 6:**
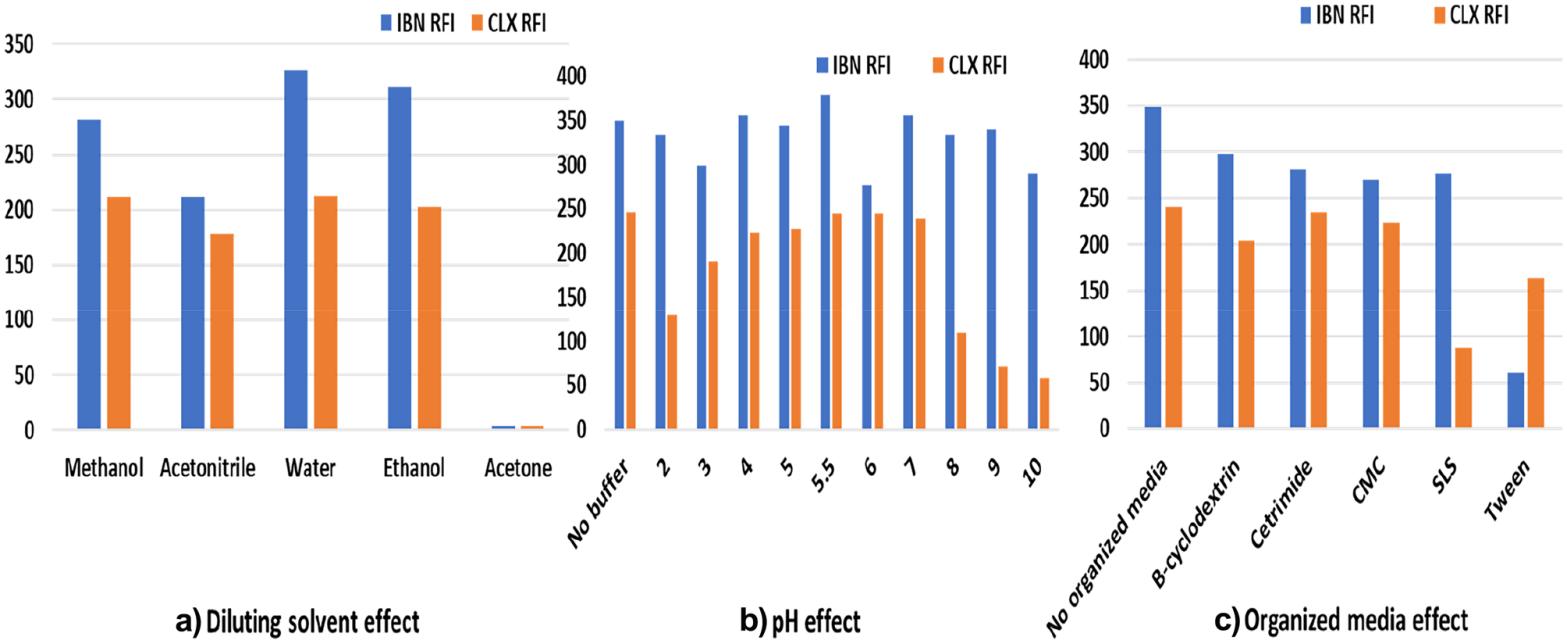
**a** Effect of diluting solvents on SF of 0.1 μg/mL IBN and 1.0 μg/mL CLX. **b** Effect of pH on SF of 0.1 μg/mL IBN and 1.0 μg/mL CLX. **c** Effect of organized media on SF of 0.1 μg/mL IBN and 1.0 μg/mL CLX

The effect of different diluting solvents, pH or organized media on SF intensities was tested to choose the optimum experimental conditions. Water, methanol, ethanol and acetone were tested as diluting solvents and it was found that water was the solvent of choice, as shown in Fig. 6a. Britton Robinson buffer with pH ranging from 2–10 was tested to choose the optimum pH. Although pH of 5.5 enhance SF for IBN, it causes slight decrease in SF of CLX. Since sensitivity of IBN is much higher than that of CLX, it was found that it is better not to use any buffer, as shown in Fig. 6b. Finally, the effect of different organized media including cetrimide, sodium dodecyl sulfate and tween-80 as surfactants and macromolecules such as carboxy methyl cellulose, and β-cyclodextrin was examined. It was found that the addition of organized media had no enhancement effect on SF as illustrated in Fig. 6c. Based on that, it was concluded that, distilled water was the best diluting solvent used, this also contributes to raising the value of the proposed method from an environmental point of view.

### Method Validation

ICH Q2R1 recommendations [24] were followed to evaluate the proposed method validity. Linearity, limit of quantitation and limit of detection, accuracy, precision and selectivity are used terms for that evaluation.

#### Linearity, Range, Limit of Quantitation (LOQ) and Limit of Detection (LOD)

After the method parameters were optimized, the proposed method was found to give a linear relation between the concentration and the resulting RSFI values over range of 0.02– 0.60 μg/mL and 0.1–5.0 μg/mL, for IBN and CLX respectively. The analytical performance of the proposed method was illustrated in Table 1. Statistical analysis of the produced data [25] showed values of the coefficients of determination with small intercepts which proves good linearity of the calibration curves. Linear regression equations of IBN and CLX are as follows:

$$\mathrm{RSFI}=48.341+1.508\times 10^{3}\;\mathrm{C}\quad \quad(\mathrm{r}=0.9999)\quad\mathrm{for}\;\mathrm{IBN}$$

$$\mathrm{RSFI}=55.241+178.382\;\mathrm{C}\quad \quad(\mathrm{r}=0.9999)\quad\mathrm{for}\;\mathrm{CLX}$$

**Table 1:** Analytical performance data for the proposed method

**Validation parameter**	**IBN** **At 227 nm**	**CLX** **At 282 nm**
**Wavelength difference**	**Δ λ = 50 nm**	
**Linearity range**	0.02–0.6 μg/mL	0.1–5.0 μg/mL
**Intercept (a)**	48.341	55.241
**Slope (b)**	1.508** × **10^3^	178.382
**Correlation coefficient (r)**	0.9999	0.9999
**S.D. of residuals (S**_**y/x**_**)**	2.465	3.601
**S.D. of intercept (S**_**a**_**)**	0.124	1.637
**S.D. of slope (S**_**b**_**)**	0.004	0.692
**Limit of detection, LOD, μg/mL**	0.272 × 10^–3^	0.030
**Limit of quantitation, LOQ, μg/mL**	0.825 × 10^–3^	0.0918

Both detection and quantitation limits were calculated using the equations reported in in ICH Q2R1 [24] and the obtained results are shown in Table 1. The obtained low LOD and LOQ indicate good sensitivity of the proposed method.

#### Accuracy and Precision

Comparison of the proposed method with the reported method [21] was performed to test the accuracy of the proposed method. No significant differences between the two methods were noticed adopting student's *t*-test and variance ratio *F*-test which confirms the accuracy of the proposed method. The results were illustrated in Table 2.

**Table 2:** Assay results for the determination of IBN, CLX and ACP in pure form by the synchronous spectrophotometry method

**Parameter**	**IBN**			**CLX**		
	**Conc. taken** **(ng/mL)**	**Conc. found** **(ng/mL)**	**% Found**	**Conc. taken** **(µg/mL)**	**Conc. found** **(µg/mL)**	**% Found**
	20.0	19.620	98.10	0.1	0.100	100.00
	30.0	29.497	98.32	0.3	0.298	99.33
	50.0	49.305	98.61	0.4	0.395	98.75
	80.0	79.882	99.85	0.5	0.499	99.80
	100.0	100.452	100.45	0.7	0.696	99.43
	200.0	201.223	100.61	1.0	0.991	99.10
	300.0	302.624	100.87	2.0	2.022	101.10
	400.0	400.380	100.10	3.0	3.029	100.97
	500.0	500.536	100.11	4.0	4.012	100.30
	600.0	597.544	99.59	5.0	4.968	99.36
**Mean ± S.D**	99.66 ± 0.987			99.81 ± 0.781		
Comparison method (n = 3) [21]						
**Mean ± S.D**	99.88 ± 0.99			99.59 ± 1.19		
***t*-value**	0.33 (2.20) *			0.39 (2.20) *		
***F*-value**	1.52 (4.26) *			2.30 (4.26) *		

***N.B.*** Each result is the average of three separate determinations

^*^The figures between parentheses are the tabulated *t* and *F* values at P = 0.05 [25]

The precision of the proposed method was assured by the accepted values of % RSD (Table 3) resulted from testing repeatability and intermediate precision. That was done by the examination of three different concentrations of each drug three times on the same day or on three successive days, respectively.

**Table 3:** Precision data for the determination of IBN and CLX by the suggested technique

**Drug**	**Conc** **(μg/mL)**	**Intra-day**			**Inter-day**		
		**Mean ± S.D**	**%RSD**	**% Error**	**Mean ± S.D**	**%RSD**	**% Error**
**IBN**	0.08	100.51 ± 0.65	0.64	0.37	99.98 ± 1.57	1.57	0.91
	0.1	100.08 ± 0.78	0.78	0.45	100.15 ± 1.64	1.63	0.94
	0.2	99.85 ± 0.83	0.83	0.48	99.53 ± 1.31	1.32	0.76
**CLX**	0.3	100.31 ± 0.88	0.73	0.42	99.79 ± 1.66	1.66	0.96
	1.0	99.55 ± 0.49	0.62	0.34	99.82 ± 1.45	1.46	0.84
	3.0	100.14 ± 0.76	0.96	0.56	99.64 ± 1.49	1.49	0.86

***N.B.*** Each result is the average of three separate determinations

#### Selectivity

**Fig. 7:**
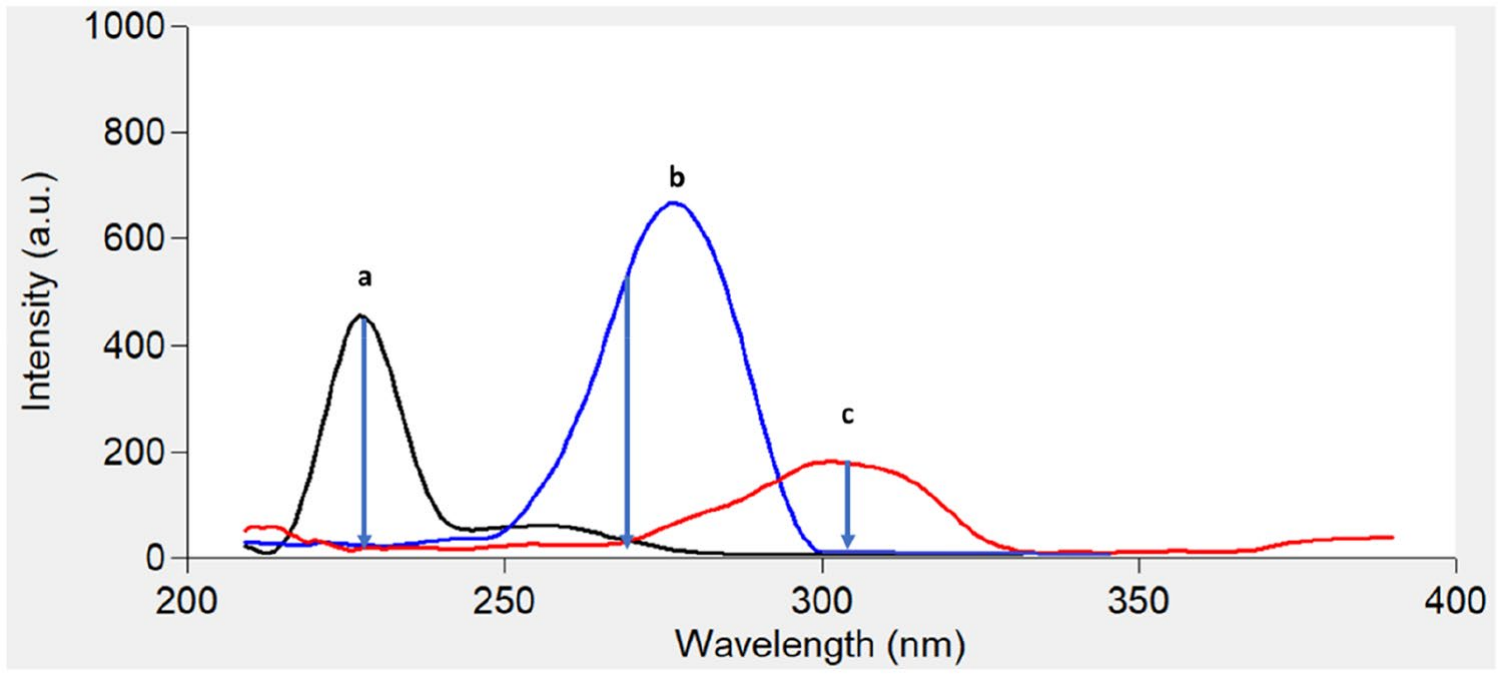
Synchronous fluorescence spectra of **a** 0.2 μg/mL IBN, **b** 3 μg/mL CLX and **c** 0.3 μg/mL ACP in ethanol

The proposed method was found to be selective, as it could clearly allow simultaneous determination of IBN and CLX without any interference. In order to test the method ability to resolve that mixture, five synthetic mixtures with the ratio of the co-formulated capsule (1:1.25) were analyzed and percent recoveries were calculated (Table 4). The produced results were compared with that of comparison method [21]. As a result of this feature, the proposed method was able to resolve the studied drugs in the biological fluid omitting the complicated matrix background. The two drugs were resolved clearly in presence of CLX degradation product as shown in Fig. 7. IBN can be determined at 227 nm, while CLX can be determined at 260 nm.

**Table 4:** Assay results for the determination of IBN and CLX in synthetic mixtures of their pharmaceutical ratio (1:1.25) by the suggested technique

**IBN/CLX ratio**	**Suggested technique**						**Comparison method** **[**21**]**	
	**Amount taken** **(µg/mL)**		**Amount found** **(µg/mL)**		**% Found**		**% Found**	
	**IBN**	**CLX**	**IBN**	**CLX**	**IBN**	**CLX**	**IBN**	**CLX**
**1:1.25**	0.1	0.125	0.098	0.127	98.00	101.60	100.32	101.14
	0.2	0.250	0.203	0.254	101.5	101.60	100.23	101.76
	0.3	0.375	0.294	0.369	98.00	98.40	98.41	101.24
	0.4	0.500	0.410	0.501	102.50	100.20		
	0.5	0.625	0.496	0.611	99.20	97.76		
	0.6	0.750	0.599	0.764	99.83	101.87		
**Mean**					99.84	100.24	99.65	101.05
** ± S.D**					1.86	1.78	1.08	0.76
***t-*value**					0.16	0.73	(2.36) *	
***F*-value**					2.93	5.44	(19.31) *	

***N.B.*** Each result is the average of three separate determinations

^*^The figures between parentheses are the tabulated t and *F* values at P = 0.05 [25]

#### Specificity

The suggested method was found to be specific as the two drugs were analyzed successfully in dosage forms and spiked human plasma without interference of either dosage forms excipients or plasma background.

### Application

#### Analysis of IBN and CLX in Laboratory Synthetic Mixtures

**Fig. 8:**
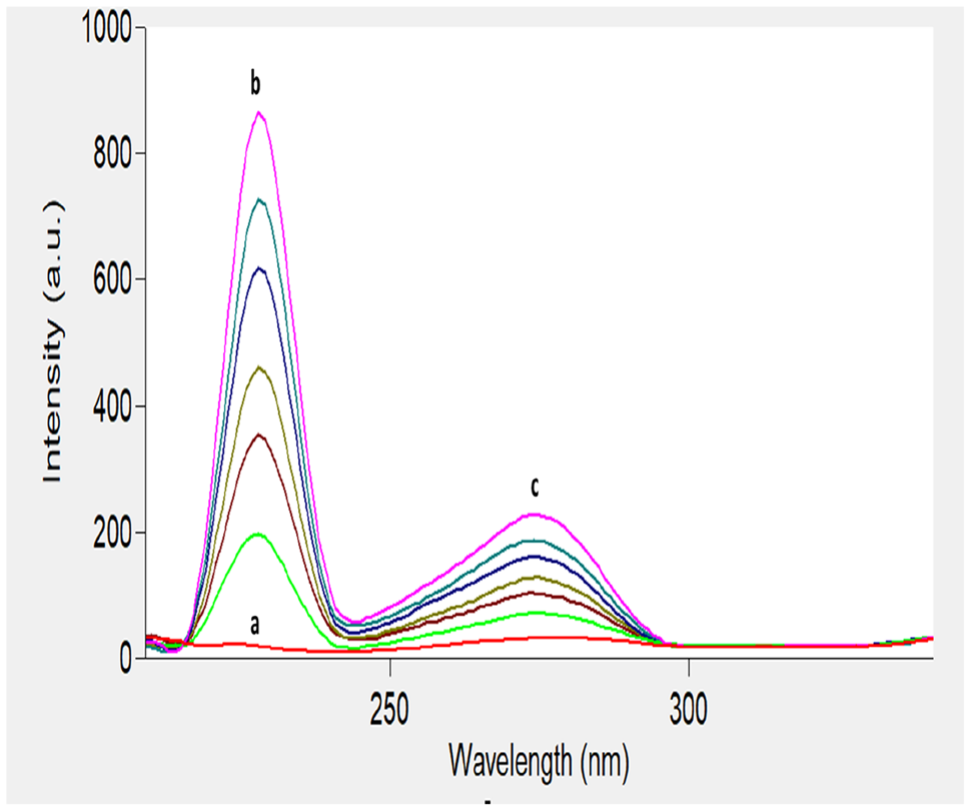
Synchronous fluorescence spectra of **a** blank, **b** IBN and **c** CLX in synthetic mixture of both

The recommended method was employed for the analysis of IBN and CLX in prepared synthetic mixtures using the ratio of (1:1.25) as in their co-formulated capsules, as presented in Fig. 8. The concentrations of the studied drugs in their synthetic mixtures could be calculated from the corresponding regression equations. The obtained results are summarized in Table 4.

#### Analysis of IBN and CLX in Their Co-formulated Capsules

**Fig. 9:**
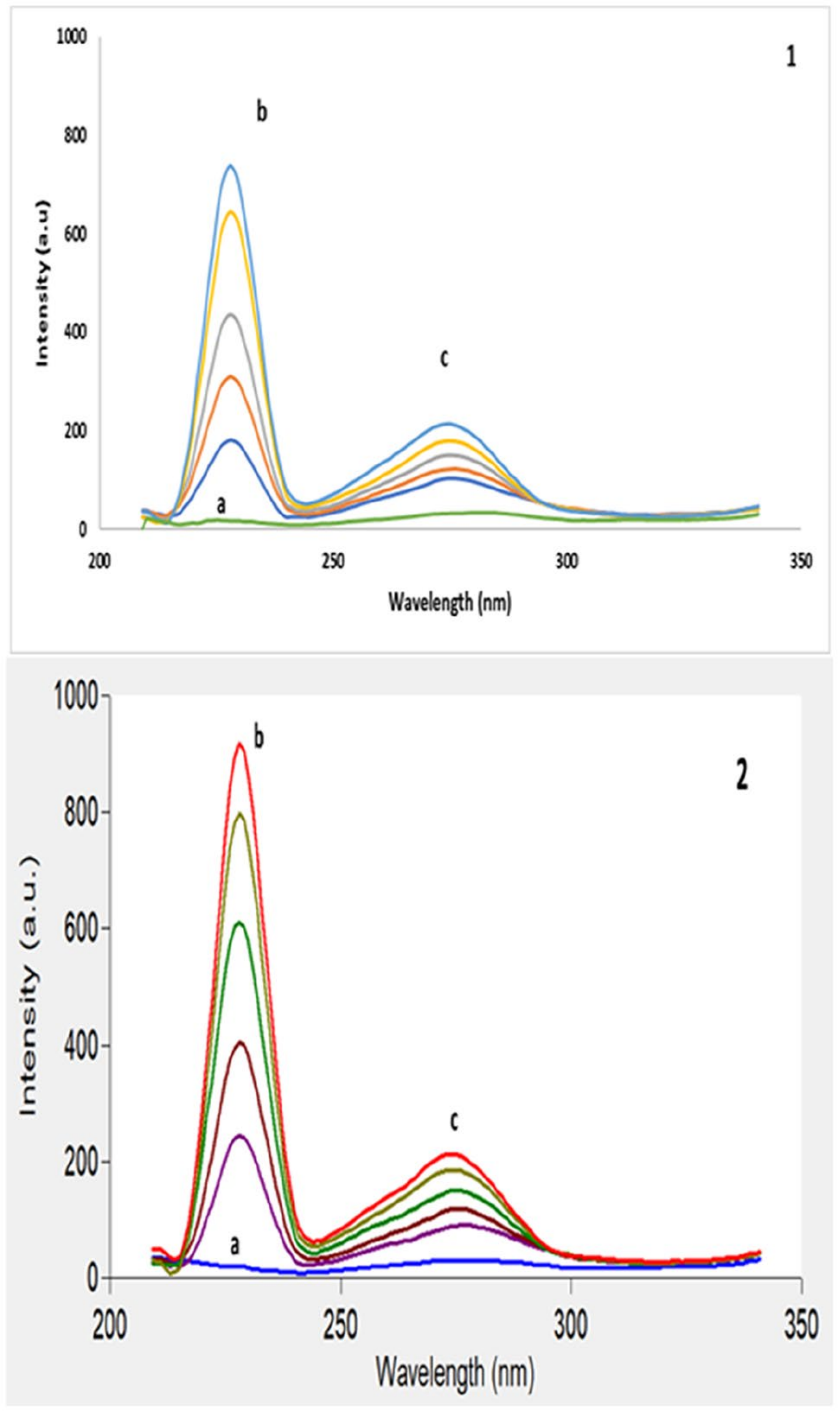
Synchronous fluorescence spectra of overlapped spectra of **a** blank, **b** IBN, and **c** CLX in (1) Mark-fast^®^ and (2) Myofen^®^ capsules

The suggested method was applied to estimate IBN and CLX in their capsules (Mark fast^®^ and Myofen^®^) and the results were satisfactory, as illustrated in Fig. 9. The common capsule additives did not interfere with the results of the proposed method, as revealed by good percentage recoveries obtained. The results of the proposed method were compared with those of the reported method [21]. As shown in Table 5, there was no significant differences between the proposed synchronous method and the comparison 2^nd^ derivative method. Moreover, the suggested method has the advantages of being greener, simpler, faster and more sensitive.

**Table 5:** Assay results for the determination of IBN and CLX in their combined capsules by the suggested technique

**Parameter**	**Reported method **^**a**^			**Comparison method** **[**21**]**
	**Drug name**	**Mark-fast**^**®**^	**Myofen**^**®**^	
**% Recovery ± SD**	**IBN**	99.91 ± 2.36	99.76 ± 2.15	99.62 ± 1.50
	**CLX**	100.10 ± 2.09	99.94 ± 1.19	99.79 ± 1.18
***t*-value **^**b**^	**IBN**	0.19	0.09	
***F*-value **^**b**^		2.47	2.05	
***t*-value **^**b**^	**CLX**	0.16	0.17	
***F*-value **^**b**^		3.11	1.00	

^**a**^ Average of five determinations

^**b**^ Tabulated value at 95% confidence limit; F = 19.25 and t = 2.44

#### Analysis of IBN and CLX in Spiked Human Plasma

The sensitivity and selectivity of the proposed method allowed the analysis of the two drugs in spiked human plasma with high accuracy. The mean peak plasma concentrations of IBN and CLX were 83 mg/L and 36.3 mg/L, respectively [23]. The results obtained in Table 6, show that the mean recoveries and the % RSD in plasma samples are 99.17 ± 2.09 and 99.99 ± 2.12 for IBN and CLX, respectively.

**Table 6:** Assay results for the determination of IBN and CLX in spiked human plasma by the suggested technique

	**Amount taken** **(µg/mL)**		**Amount found** **(µg/mL)**		**% Found**	
**Spiked human plasma**	**IBN**	**CLX**	**IBN**	**CLX**	**IBN**	**CLX**
	0.2	0.25	0.097	0.251	96.48	100.80
	0.3	0.375	0.207	0.38	100.99	101.06
	0.4	0.5	0.297	0.481	101.98	96.20
	0.5	0.625	0.401	0.63	99.60	100.96
	0.6	0.75	0.499	0.758	99.49	100.93
**Mean**					99.17	99.99
** ± S.D**					2.08	2.12
**%RSD**					2.09	2.12
**%Error**					0.93	0.95

***N.B.*** Each result is the average of three separate determinations

#### Evaluation of Greenness

Although the studies focused on eliminating the waste and adopting ecofriendly and sustainable methods [26–28] started in 1995, they were not assessed by the analytical society. One of the priorities of green analysis is to reduce the use of harmful substances without affecting the efficiency of the methods performance [29–31].

Recently, green analysis as well as indexing the method greenness have become very important. Indexing the method greenness allows the possibility of ranking the methods according their greenness which was found to be very helpful [32, 33]. Four assessing methods were employed to assess the greenness of the recommended technique and compare it with the reported one.

First, National Environmental Methods Index (NEMI) [32] was applied on the proposed and reported method. NEMI is a tool using greenness profile and regarded as one of the first methods to appear. Table 7 shows that the proposed method achieves the four criteria of the greenness profile and is greener than the reported method according to NEMI profile. Water and ethanol are neither classified as PBT nor hazardous by the EPA’s Toxic Release Inventory [26, 27], the pH is not corrosive and the waste is less than 50 g / run.

**Table 7:** Comparison between the suggested and reported methods

**Parameters**	**Reported method** **[**21**]**	**Proposed method**
**Linearity range**	0.1-1.6, 0.2-4 μg/mL	0.02-0.6, 0.1-5 μg/mL
**Solvent and Reagents**	Methanol Borate buffer Citrate solution Phosphate buffer Chloroform Anhydrous sodium sulphate	Ethanol Water Methanol
**Method of Assay**	Second devastative synchronous fluorescence at Δλ=60 nm	Direct synchronous fluorescence at Δλ=50 nm
**NEMI**		
**GAPI**		
**Eco-Scale**	Acceptable green analysis 100-25=75	Excellent green analysis 100-8=92
**AGREE**		

Second, GAPI (Green Analytical Procedure Index) [33] was also applied on the proposed and reported methods. The green assessment GAPI profiles for the proposed and reported method are presented in Table 7.

Additionally, analytical Eco-scale was utilized for evaluating the proposed and reported methods, as represented in Table 7. The proposed method’s score is 95 which idicates an excellent green methodology (the closer the score to 100, the greener the method) [34].

Finally, the greenness of the proposed method was investigated using AGREE-Analytical GREEnness Metric Approach and software through evaluating 12 parameters of green analytical aspects. Table 7 represents the twelve parameters with different colors ranging from dark green to orange based on information reported by Francisco Pena-Pereira et al. [35]. The score was 0.84 which indicate the method greenness (the closer the score to 1.0 the greener the method).

As described previously by the four assessment tools, it was concluded that the suggested technique has an environmental advantage over the reported methods, and thus it could be employed for the routine analysis of IBN and CLX without affecting the environment.

## Conclusion

The proposed method presents a one step green spectrofluorimetric method for the concurrent assay of IBN and CLX binary mixture. The analysis was performed directly without any separation or extraction steps. Moreover, it is very simple, rapid and economic compared to other techniques that require complicated instruments or steps. The suggested technique showed high sensitivity that made it suitable for the analysis of the two drugs in human plasma. The method greenness was assessed using four tools including NEMI, GAPI, analytical Eco-Scale and AGREE. The developed method was fully validated according to ICH guidelines.

## Data Availability

All the data generated or analysed during this study are included in this article.
